# Annotations

**Published:** 1896-09-12

**Authors:** 


### ANNOTATIONS.

### Medical Coercion.

WHENEVER medical men have attempted to bind themselves together with the object of making medical practice less intolerable, "the public," said Dr. Mitchell Banks the other day to a northern branch of the British Medical Association, "have immediately called us a 'trade union.' If that be so," he continued, "then let us be a trade union, and let us be a trade union to some purpose." "Show me a greater trade union than the Bar," said the indignant doctor. "A barrister will take cases in two courts, and only attend one of them, pocketing the fees for both cases all the same. If a doctor, in like manner, should take fees in advance for midwifery cases, and then attend only just so many as he conveniently could, would there not be a howl of indignation in a neighbouring court of justice, and would not the wicked practitioner be compelled to disgorge his fees for those cases he had been unable to attend? As for doctors," said Dr. Banks, "Vestries and Boards of Guardians screw them down to the last penny, whilst Corporations and County Councils remunerate the services of their medical officer of health as they would the services of a cabman." All this, Dr. Banks thinks, is the fault of a certain class of doctors; and we agree with him. How would he mend matters? He would coerce the "undersellers" by putting them beyond the pale of professional recognition; and here again we agree with him, if we may define what we mean by "undersellers." Nothing is more curious than the low estimate that some men put upon their labour, and if men choose to work all day, and too often all the night, for small reward, we do not feel inclined to say that therefore they should be ostracised so long as their work is good, and herein we differ from the trades unionism of the trades. But when men offer to work at fees at which we know that the services rendered cannot be honest services, then there need be no hesitation whatever in applying to them the sharpest form of coercion which the law permits.

### The Microbe at Swansea.

AN important discussion is being carried on by some members of the Managing Committee of the Swansea Hospital, which, if humour be not very dreadful in such a connection, appears to have its humorous side. The medical staff of the hospital have presented a report, the point of which is that a new operation theatre is imperatively necessary; and the report insists that the theatre must be proceeded with on the instant, because "micro-organisms" abound in the present theatre, and because, therefore, "it is clearly shown that in inviting the poor into the hospital for the treatment of wounds and the performance of surgical operations, we invite them to incur risks which, if they could afford the expense, they would not incur in their own homes." Upon this, a lay member of the committee, Mr. D. Sugrue, deeply concerned about ways and means, as is the duty of such members, makes reply that the finances of the institution are in a depressed condition; that, moreover, the microbes notwithstanding, the proportion of surgical cures at the hospital is very high; and that, in a word, the microbe theory is brought in for the purpose of "scotching" opposition and securing a new operation theatre, whether there be proved necessity or not. The question of real urgency is this: Is the operation theatre in such a condition that a thorough cleansing and "restoration" cannot make it again available? This point we think ought to be settled; and if it is decided that the theatre cannot be restored to a sanitary condition, and that in consequence of its present state the recovery of the patient is jeopardised, then we have no doubt that Mr. Sugrue and all other opponents whom he may for the time represent will join heartily in the demand for a new theatre, and proceed to get together the money in the most effectual way they can.

### Milk for Infants.

IN a little pamphlet on Milk and Infantile Disease Dr. Henry Ashby tells us again the old familiar tale as to the impurities contained in the milk of commerce. People, however, are by no means backward in declaring that all this outcry about microbes is mere faddy nonsense, and that although these things have been going on for thousands of years the human race survives. It must then be pointed out that things as they now exist have not been going on for thousands of years. The old relationship between man and the domestic animals, according to which they have grown up and developed alongside of one another, have been personal and individual. The milk of the man's own cow has gone to the rearing of his own family, and at the most a farmer's milk has been distributed among his customers. The wholesale collection of milk from large areas, the mixing of milk from hundreds of cows, and its distribution to thousands of consumers, its transport by rail for hundreds of miles, its preservation by means of ice and chemical substances, so that it will keep for the long periods which must intervene between the cow and the kitchen, the separation of the cream, and the local adulteration of milk maybe from one county with "skim" from another—all these are things of recent times, and when sanitarians demand new remedies, it is that they may fight new evils. Moreover, it is not true that in despite of all these evils the race thrives. We, the grown men, thrive perhaps—but then we do not take the milk. But as to the children who do take it, undoubtedly they die; the difference in the mortality of mother-fed and cow-fed babies being enormous. To remedy this state of affairs Dr. Ashby advocates a reform of the milk trade in two directions; one, that in which the Milk Supply Company in Copenhagen is working, viz., the supply of pure bottled milk, unsterilised but uncontaminated; the other the line taken by the "milk laboratories" which have been established in some cities in America, in which not only is the most scrupulous care taken in the collection of the milk, but this is then separated and recombined in such proportions as may be ordered by the physician as suitable for the individual case, and is then delivered to the customer bottled and sterilised or pasteurised, as the case may be. We can have no doubt that the ultimate solution of the milk problem will be in the direction of cleanly collection and distribution in glass bottles. This is already done in a half-hearted sort of way, probably as much being spent in distribution as on the milk itself, owing to the widely-scattered clientele. If people could be assured that the whole week's milk might be bought at once, the milk supply, just the same as that of aerated waters, would fall into the hands of the grocers, and there can be no doubt that the price would be reduced. The cost of milk lies in the waste thereof, and in the expense of distributing it twice a day.

